# High-fat diet modifies expression of hepatic cellular senescence gene p16(INK4a) through chromatin modifications in adult male rats

**DOI:** 10.1186/s12263-018-0595-5

**Published:** 2018-03-14

**Authors:** Xiyuan Zhang, Guanying Bianca Xu, Dan Zhou, Yuan-Xiang Pan

**Affiliations:** 1 Pediatric Oncology Branch (POB), National Cancer Institute (NCI), National Institute of Health (NIH), Bethesda, MD 20892 USA; 2 Department of Food Science and Human Nutrition, University of Illinois Urbana-Champaign, 461 Bevier Hall, MC-182, 905 South Goodwin Avenue, Urbana, IL 61801 USA; 3 Division of Nutritional Sciences (DNS), University of Illinois Urbana-Champaign, 461 Bevier Hall, MC-182, 905 South Goodwin Avenue, Urbana, IL 61801 USA; 4 Hongqiao International Institute of Medicine, Shanghai Tongren Hospital/Faculty of Public Health, Shanghai Jiao Tong University School of Medicine, Shanghai, 200025 China; 5 Illinois Informatics Institute, University of Illinois at Urbana-Champaign, 461 Bevier Hall, MC-182, 905 South Goodwin Avenue, Urbana, IL 61801 USA

**Keywords:** High-fat diet, p21(Cip1), Fatty liver, Hepatic cellular senescence, Chromatin modification

## Abstract

**Background:**

Liver is the crucial organ as a hub for metabolic reactions. p16(INK4a) is a well-established cyclin-dependent kinase (CDK) inhibitor that plays important role in the molecular pathways of senescence, which lead to irreversible cell cycle arrest with secretion of proinflammatory cytokines and mitochondrial dysfunction. This study tested the hypothesis that cellular senescence regulated by p16(INK4a) is associated with high-fat diet in adult male rats.

**Methods:**

Sprague Dawley rats were fed a high-fat (HF) diet or a control (C) diet for 9 weeks after weaning. At 12 weeks of age, liver samples of male rats were collected to investigate the key genes and liver physiological status.

**Results:**

Both mRNA and protein expression level of cellular senescence marker, p16(INK4a), was increased significantly in HF group when compared to C group. A decrease of tri-methylated histone H3 lysine 27 (H3K27Me3) in the coding region of p16(INK4a) was observed. On the other hand, mRNA and protein expression of another inhibitor of cyclin-dependent kinase, p21(Cip1), was decreased significantly in HF group; however, no significant chromatin modification was found in this gene. Histological analysis demonstrated hepatic steatosis in HF group as well as severe fat accumulation.

**Conclusions:**

Our study demonstrated that HF diet regulated cellular senescence marker p16(INK4a) through chromatin modifications, which may promote hepatic fat accumulation and steatosis.

## Background

Dietary factors play pivotal role in modulating liver function and physiology as well as in the development of hepatic cells: a high-fat (HF) diet can increase hepatic lipid content and plasma insulin concentration, causing insulin resistance in obese animals [1–3], while maternal high-fat diet leads to altered regulation of liver development in offspring through DNA methylation, which may cause early hepatic dysfunction [4]. Several animal models have been reported to establish the association between high-fat diet and the physiological changes in the liver [5–10].

Among the annotated genes using the Gene Oncology database, cell cycle control genes were the second largest group of altered genes associated with HF diet-induced changing [11], and this link between HF diet and cell cycle regulation has also been reported in other studies using either cell or animal models [4, 12, 13]. p16(INK4a) is a biomarker of cellular senescence and aging [14, 15], and its expression has been linked to replicative hypofunction in many types of cells, including hematopoietic progenitors [16], lymphocytes [17, 18], neural stem cells [19], and pancreatic beta cells [20]. Pervious study has shown that high-fat diet induced increasing level of p16(INK4a) transcription rate in an obese rat model, which was associated with a higher acetylation levels of histone H4 and lower methylation level of histone H3 lysine 27 in p16(INK4a) promoter and coding region [21]. p21(Cip1), on the other hand, belongs to the family of CDK inhibitor proteins and is a universal inhibitor of CDKs which include cyclin D-, E-, and A-dependent kinases [22, 23]. Upregulation of p21(Cip1) protein was proved to be involved in impaired regeneration of fatty livers in obese mice [4]. Although literature indicates that p16(INK4a) and p21(Cip1) alteration may not be necessary for either exogenous or endogenous liver carcinogenesis [24], it remains unclear how high-fat diet affects expressions of these CDK inhibitors and how the expression changes affect liver function as well as hepatic physiology.

As briefly mentioned before, the expressions of p16(INK4a) gene is regulated through epigenetic modifications. Hypermethylation of DNA in promoter CpG islands is associated with the transcriptional silencing of p16(INK4a) gene, and the di-methylation of histone H3 lysine 9 (H3K9) correlates with the DNA methylation status in colon cancer development [25]. High-fat diet was proved to alter DNA methylation in genes related to liver lipid metabolism and hepatic steatosis: for example, *Ndufb9*, or NADH dehydrogenase 1 beta subcomplex 9, was found hyper-methylated in CpG sites in the HFD group, which downregulates its expression [26]. However, few studies have been done to investigate the role of dietary high fat in regulation of p16(INK4a) expression through epigenetic modifications.

This study was designed to investigate the expression and chromatin modifications of CKIs p16(INK4a) and p21(Cip1) in adult male rat model induced by high-fat diet and its effects on hepatic cellular senescence as well as liver physiology. We hypothesized that high-fat diet regulates hepatic cellular senescence in male adult rats by regulating p16(INK4a) and p21(Cip1) expression through chromatin modifications, which may promote hepatic fat accumulation and steatosis.

## Methods

### Animal study

Sprague Dawley rats were fed a high-fat diet (HF) or a control (C) diet for 9 weeks after weaning. All information regarding the diets (Research Diets, Inc., New Brunswick, NJ) is listed in Table 1. Animals were individually housed in standard polycarbonate cages in a humidity- and temperature-controlled room on a 12-h light-dark cycle and had free access to food and water. At 12 weeks of age, the animals were sacrificed and the left lobe of the liver was collected, rapidly frozen in liquid nitrogen, and stored in − 70 °C for future use. We confirm that all applicable institutional and governmental regulations regarding the ethical use of animals were followed during this research (University of Illinois Institutional Animal Care and Use Committee approval #09112).

**Table 1:** Diet formula

Diet formula	C	HF
Total energy, kJ/g	16.75	19.68
Protein, g/kg	20	24
Carbohydrate, g/kg	64	41
Fat, g/kg	7	24
Ingredient, g/kg		
Casein	200	200
l-Cystine	3	3
Corn starch	437.2	72.8
Maltodextrin	100	100
Sucrose	102	172.8
Cellulose	50	50
Soybean oil	25	25
Lard	47	177.5
Mineral mix^a^	10	10
DiCalcium phosphate	13	13
Calcium carbonate	5.5	5.5
Potassium citrate	16.5	16.5
Vitamin mix^b^	10	10
Choline bitartrate	2	2
Yellow dye #5	0.025	N/A
Red dye #40	0.0025	0.05

The diet formula is provided by http://www.researchdiets.com/

^a^Mineral mix (AIN-93): (research diets) product no. S10026 for rodents

^b^Vitamin mix (AIN-93): (research diets) product no. V10001 for rodents

### RNA isolation and cDNA synthesis

Frozen liver samples (50–100 mg) were ground using mortar and pestle with liquid nitrogen, and total RNA was extracted with TRI reagent (Sigma, St. Louis, MO). RNA samples were incubated with DNase Ι (Roche, Mannheim, Germany) at room temperature for 20 min, followed by 10 min in 90 °C heat block to prevent contamination of genomic DNA. High-capacity cDNA Reverse Transcription Kit (Applied Biosystems, Foster City, CA) was used for reverse transcription of 2 μg of total RNA. Reverse transcription was performed in a 2720 Thermal Cycler (Applied Biosystems) with 20 μL reaction volume with the following program: heated at 25 °C for 10 min, incubated at 37 °C for 2 h, and then heated at 85 °C for 5 s. The 20 μL of final product was diluted to 400 μL for 5 ng/μL using RNase free water (Fisher, Fair Lawn, NJ) and stored at − 20 °C.

### Real-time quantitative PCR

To measure the relative transcriptional level of target genes, qPCR was performed in a 96-well plate using a 7300 real-time PCR System (Applied Biosystems). Five microliters of cDNA, with the final concentration of 5 ng/μL, was mixed with 10 μL SYBR Green master mix (Quanta Biosciences Inc., Gaithersburg, MD) and 1 μL of each 5 nmol/L primer (forward and reverse). The reaction was incubated using the following program: 95 °C for 10 min, followed by 35 cycles of 95 °C for 15 s and 60 °C for 1 min. High efficiency of the machine and the presence of a unique product were ensured by checking that the slope of the standard was in the range of − 3.3 ± 0.3 and the *R* square to be larger than 0.98. Kinetic analysis was conducted to detect the exponential phase of amplification in each well with 25 ng template cDNA. mRNA level of ribosomal protein L7a was utilized as the internal control. In order to determine the gene transcription rate, primers were designed to amplify a region including both exon and intron of that gene. The relative expression level of this region can indicate the pre-mRNA rate of the gene and further present the transcription rate. Primers used for qPCR were designed using Vector NTI software (InforMax Inc., Frederick, MD), and all the primers used for real-time PCR are listed in Table 3.

### Triacylglycerol and non-esterified fatty acid assay in plasma and liver tissues

One hundred milligrams of frozen liver samples were ground using a mortar and pestle with liquid nitrogen and mixed with 0.3 mL saline (0.9% *w*/*v* NaCl). Homogenized samples were quickly frozen in liquid nitrogen and kept in − 70 °C until analysis. The samples were quickly thawed in 37 °C and diluted five times to 1.5 mL. Twenty microliters of the diluted samples was incubated with 20 μL 1% deoxycholate in 37 °C for 5 min, and 10 μL of the samples was analyzed via the Thermo Infinity Triglycerides Liquid Stable Reagent (Thermo Fisher Scientific, Rockford, IL) following company protocol and using a commercially available standard reference kit (Verichem Laboratories, Providence, RI). Lowry assay was performed to determine the protein concentration, which was used to normalize the triacylglycerol (TAG) concentration in the liver. To determine plasma TAG levels, plasma samples were thawed on ice and analyzed accordingly following company protocol as for liver samples. Non-esterified fatty acid concentration in the liver was determined using a commercially available kit (HR-2 Series, Wako Diagnostics, Richmond, VA).

### Liver histology

Frozen liver samples from offspring were embedded in O.C.T. compound (Gentaur, Kampenhout, Belgium) prior to sectioning. All sections were then stained with hematoxylin and eosin (H&E) or oil red O (ORO, Newcomer Supply, Middleton, WI) and evaluated for steatosis, fat accumulation, and inflammation, by a pathologist blind to the identity of the experimental groups.

### Chromatin immunoprecipitation (ChIP)

To determine the specific chromatin modification and transcription factor binding, ChIP analysis was employed according to a modified protocol [27]. Two hundred milligrams of frozen liver samples were ground using a mortar and pestle with liquid nitrogen and washed with PBS. The samples were resuspended in PBS and cross-linked in 1% formaldehyde for 10 min at room temperature. After centrifugation, the pellet was resuspended in nuclei swelling buffer (5 mmol/L Pipes [NaOH] pH 8.0, 85 mmol/L KCl, 0.5% NP40) containing protease inhibitor and phosphorylation inhibitor. The nuclei were lysed in SDS lysis buffer (50 mol/L Tris-HCl pH 8.1, 10 mmol/L EDTA, 1% SDS) containing protease inhibitor and phosphorylation inhibitor. The chromatin was sonicated (Fisher Scientific model 100 Sonic Dismembrator, Pittsburgh, PA) on ice with six bursts for 40 s at power setting 5 with 2 min cooling between each burst. After removing the cell debris by centrifuging the sonicated product at 13,000 rpm in 4 °C for 10 min, sheared chromatin was diluted in ChIP Dilution Buffer to 10 mL to perform 10 immunoprecipitations (IPs). One milliliter of the diluted lysate was incubated overnight on a hematology mixer (Model 346, Fisher Scientific) with 2 μg of primary antibodies at 4 °C (all the antibody information is listed in Table 2). Sixty microliters of pre-blocked salmon sperm DNA/protein G agarose beads (60 μl, 50% slurry; Upstate Biotechnology, Lake Placid, NY) was then added to each chromatin sample, followed by 2 h of incubation at 4 °C. The mixture was then centrifuged at 2000 rpm for 1 min at 4 °C. Supernatant of normal rabbit IgG was saved as input control. The pellets containing immunoprecipitated complexes were washed sequentially with 1 mL of low-salt solution (0.1% SDS, 1% triton X-100, 2 mM EDTA, 20 mmol/L Tris-HCl pH 8.0, 150 mmol/L NaCl), high-salt solution (0.1% SDS, 1% triton X-100, 2 mM EDTA, 20 mmol/L Tris-HCl pH 8.0, 500 mmol/L NaCl), and LiCl solution (0.25 mol/L LiCl, 1% NP40, 1% sodium deoxycholate, 1 mM EDTA, 10 mmol/L Tris-HCl pH 8.0) and twice with TE (pH 8.0). Antibody/protein/DNA complexes were eluted from Protein G beads by adding twice 250 μL of the elution buffer (1% SDS and 50 mmol/L NaHCO_3_) followed by shaking at 37 °C for 15 min at 300 rpm and flash spinning down at room temperature. The combined supernatants were incubated at 65 °C for 5 h with 20 μL 5 mol/L NaCl and 1 μg of RNase A (Qiagen, Hilden, Germany) to reverse the formaldehyde cross-linking and release the DNA fragments. Samples were then treated with proteinase K (Sigma) at 37 °C for 1 h to remove protein. DNA was purified with a DNA miniprep system (Qiagen). Five percent of immunoprecipitated DNA was used for real-time PCR reaction to detect promoter and coding regions of the p16(INK4a) and p21(Cip1) genes. Primers were designed using Vector NTI software (InforMax Inc., Frederick, MD) and are listed in Table 3.

**Table 2:** Antibodies used in ChIP

Antibody	Species	Company	Catalog no.
IgG	Rabbit	Santa Cruz	sc-2027
H3Ac	Rabbit	Millipore	06-599
H4Ac	Rabbit	Upstate	06-866
H3K9Me2	Rabbit	Millipore	07-030
H3K9Me3	Rabbit	Upstate	07-442
H3K27Me3	Rabbit	Upstate	07-449
Pol II		Millipore	05-623

**Table 3:** Primers, locations, and functions

Gene	Sequence	Function	Ensembl ID
p16(INK4a)	Forward (p16(INK4a) + 12F) 5′-TGCAGATAGACTAGCCAGGGGA-3′ Reverse (p16(INK4a) + 76R) 5′-CTTCCAGCAGTGCCCGCA-3′	mRNA	ENSRNOT00000066011
p16(INK4a)	Forward (p16(INK4a) − 61F) 5′-ACTGGGCGGGCACTGAATCTC-3′ Reverse (p16(INK4a) + 101R) 5′-TCCGGGGCGTTTGGTGAAG-3′	ChIP promoter	
p16(INK4a)	Forward (p16(INK4a) + 5829F) 5′-CGGGTCACCGACAGGCATAA-3′ Reverse (p16(INK4a) + 5889R) 5′-TTGGACCACCCATGCTCACC-3′	ChIP coding and transcription rate	
p16(INK4a)	Forward (p16(INK4a) + 7947F) 5′-TCCGAGTCCCCATCTGTGACTGT-3′ Reverse (p16(INK4a) + 8016R) 5′-TGGAGGATGGATTGGAGCACC-3′	ChIP downstream	
p21(Cip1)	Forward (p21(Cip1) + 5100F) 5′-CCGAGAACGGTGGAACTTTGAC-3′ Reverse (p21(Cip1) + 5171R) 5′-GAACACGCTCCCAGACGTAGTTG-3′	mRNA and ChIP	ENSRNOT00000000628
p21(Cip1)	Forward (p21(Cip1) − 320F) 5′-GACAGACCTGTAAACGCTCAAC-3′ Reverse (p21(Cip1) − 233R) 5′-ACACCTGCTTGTCACGCA-3′	ChIP promoter	
p21(Cip1)	Forward (p21(Cip1) − 5200F) 5′-GGCTCACTTACACTTCCCAG-3′ Reverse (p21(Cip1) − 5125R) 5′-GGTTTCATTGGCTGGAGC-3′	ChIP upstream	
p21(Cip1)	Forward (p21(Cip1) + 6388F) 5′-AAAACGGAGGCAGACCAG-3′ Reverse (p21(Cip1) + 6450R) 5′-TCCCGCAGTATCTTGCCT-3′	Transcription rate	
L7a	Forward (L7a + 67F) 5′-GAGGCCAAAAAGGTGGTCAAT-3′ Reverse (L7a + 127R) 5′-CCTGCCCAATGCCGAAGTTCT-3′	mRNA	ENSRNOT00000006754

### Immunofluorescent staining

Unfixed, frozen colon sections were embedded in Tissue-Tek OCT compound (VWR, Radnor, PA) and cut to 8-μm-thick sections using Leica CM3050 S cryostat (Leica Microsystems, Inc., IL) at − 24 °C; sections were stored in − 80 °C before immunofluorescence staining. When conducting an immunofluorescent staining, slides were first washed in 1× PBS and fixed in 4% formaldehyde. Then, slides were blocked with blocking buffer (1× PBS/5% normal goat serum/0.05% Triton X-100) and incubated with p21 antibody (F-5) Alexa Fluor 488 (1:50, sc6246 AF488, Santa Cruz Biotechnologies, Dallas, TX) and anti-CDKN2A/p16INK4a antibody (1:100, ab211542, Abcam, Cambridge, UK) at 4 °C overnight. After primary incubation, slides were washed again with 1× PBS and then incubated with Goat anti-Rabbit IgG (H+L) Highly Cross-Adsorbed Secondary Antibody, Alexa Fluor 647 (1:100, Thermo Fisher Scientific, Eugene, OR) at room temperature for 1 h. All slides were then rinsed in 1× PBS again and incubated with DyLight 554 Phallioidin (1:50, Cell Signaling Technology, Danvers, MA) and Hoechst 33342 (Thermo Fisher Scientific, Eugene, OR), respectively, for 15 min in dark at room temperature. After incubation, slides were washed with 1× PBS, mounted with Prolong-Gold Antifade Reagent (Molecular Probes by Life Technologies, Carlsbad, CA), and dried in dark overnight at 4 °C. Pictures were taken using the Confocal LSM 700 microscope (Carl Zeiss Microscopy, LLC, United States) with Zen software (Carl Zeiss AG, Ontario, CA) at magnification of × 10. To quantify protein expression, five 350 μm × 350 μm captured pictures in each group were analyzed. ImageJ Software (NIH, Bethesda, MD) was applied to separate the nucleus spheres and p16 or p21 protein spheres by channel (blue, red and green) and to measure sphere areas. Protein expression is calculated by the protein spheres (μm^2^) ratio to nucleus sphere area (μm^2^).

### Statistical analysis

Results are presented as mean ± SEM (animal numbers of specific experiments are descripted in the figure legends). Comparison of food intake and body weight during treatment period and chromatin modifications at p16 and p21 genes between HF and C groups were performed by one-way analysis of variance (ANOVA) using proc GLM program in SAS v. 9.1 (SAS Institute, Cary, NC). Comparison of other physiological outcomes, mRNA expression, and protein expression were performed by two-trailed *t* test using proc GLM program in SAS v. 9.1 (SAS Institute, Cary, NC). Significance (*) was set at *p* < 0.05 level.

## Results

### Physiological outcomes

#### Food intake and body weight

**Fig. 1:**
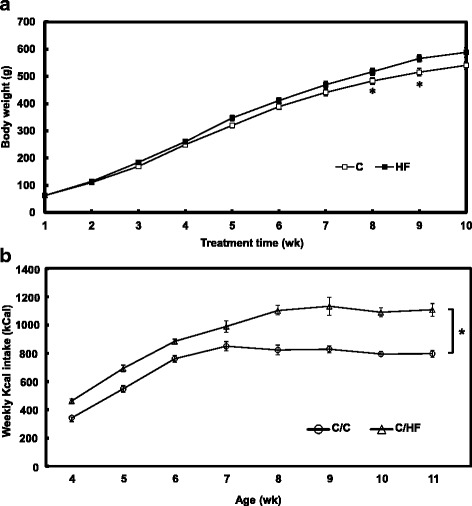
Physiological outcome of rats fed high-fat diet and control diet. **a** Effect of different diets on body weight of C and HF rats through 9 weeks of feeding. Values are mean ± SEM, *p* < 0.05*. **b** Energy intake graph of rats fed with different diets: control (C, *n* = 10) diet, and high-fat (HF, *n* = 10) diet. Values are mean ± SEM. *p* < 0.05*

The body weight (g) of rats fed HF diet was significantly higher than those fed C diet after 7 weeks of feeding (Fig. 1a). Energy intake (kCal) in HF group was significantly different from that in the C group (Fig. 1b).

#### Plasma and liver triacylglyceride (TAG) and non-esterified fatty acid (NEFA)

**Fig. 2:**
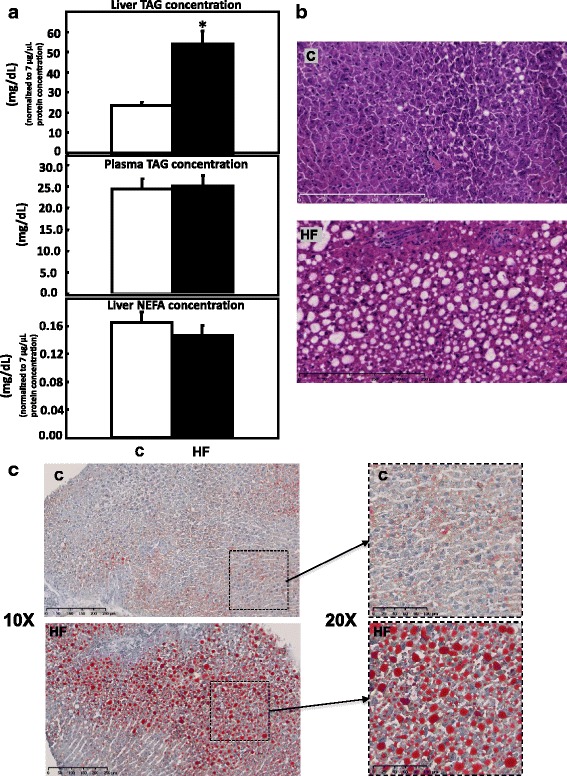
Liver histological outcome rats fed HF and C diets. **a** Triglyceride (TAG) concentration in plasma and liver, non-esterified fatty acid (NEFA) concentration in the liver. Data are presented in arbitrary units as relative liver TAG or NEFA abundance normalized to protein abundance. Values are mean ± SEM, *P* < 0.05*. **b** H&E stain of representative liver sections (× 20). **c** ORO stain of representative liver sections (× 10 left and × 20 right)

Plasma TAG levels were not different between the HF and C groups, while liver TAG significantly increased in the rats of HF group when compared to the C group. No significant difference was observed in liver NEFA level between C and HF groups (Fig. 2a).

#### Hematoxylin and eosin (H&E) stain and oil-red-O (ORO) stain

Photomicrographs of liver sections stained with hematoxylin and eosin are shown in Fig. 2b. As expected, based on observations made by previous studies [6, 28–31], comparing to liver tissue collected from C group, liver tissue from HF group showed development of hepatic steatosis and extensive degenerative ballooning, which was predominantly microvascular and involved zone 3 (perivenular) hepatocytes [21]. ORO staining suggests that liver tissue collected from HF group has increased fat accumulation with larger lipid droplets (Fig. 2c). Together, these observations indicated that high-fat diet triggered significant histological changes in the liver, increased hepatic lipid accumulation, and hence induced fatty liver.

### Cellular senescence parameters

#### Gene expression

**Fig. 3:**
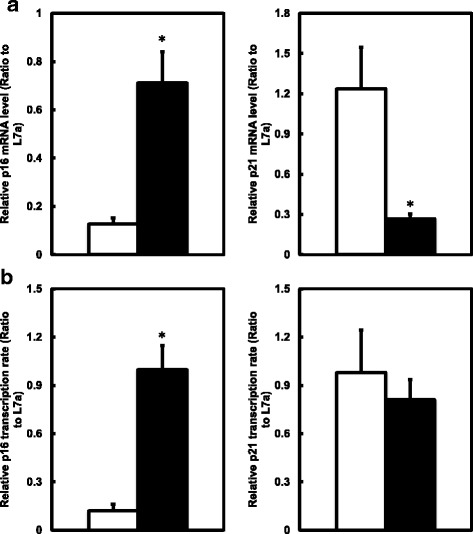
Effects of different diets on hepatic cellular senescence genes in male rats. **a** p16(INK4a) and p21(Cip1) mRNA level in C and HF rats as determined by quantitative real-time PCR. Data are presented in arbitrary units as relative mRNA abundance normalized to rL7a transcript abundance (*n* = 10). Values are mean ± SEM, *P* < 0.05*. **b** p16(INK4a) and p21(Cip1) transcription rate under different maternal diets in male offspring rats as determined by quantitative real-time PCR (*n* = 10). Values are mean ± SEM, *P* < 0.05*

mRNA level of p16(INK4a) gene, as a marker of cellular senescence, increased significantly in livers of rats in HF group when compared to the C group, while the mRNA level of p21(Cip1), another inhibitor of cyclin-dependent kinase, was significantly lower in HF group than in C group (Fig. 3a). Transcription rate of p16(INK4a) gene was accordingly significantly increased in HF group, while the transcription rate of p21(Cip1) gene did not change between the two groups (Fig. 3b).

#### Protein level of p16 (INK4a) and p21 (Cip1) by immunofluorescent staining

**Fig. 4:**
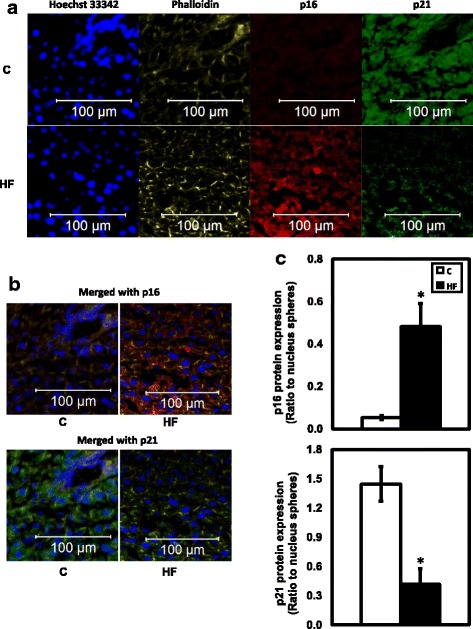
Confocal immunofluorescent pictures (× 10) of rat livers and protein expression analysis. **a** Single channel pictures of stained nucleus (blue), actin filament (yellow), p16 INK4a antibody Alexa Flour 647 conjugated (red), and p21 Cip1 antibody Alexa Flour 488 conjugated (green). **b** Merged pictures (p16 INK4a/actin/nucleus and p21 Cip1/actin/nucleus, respectively) are selected and presented respectively to visualize protein quantification and localization. **c** p16 (INK4a) and p21 (Cip1) quantified protein level in C and HF rats as determined by immunofluorescence signal intensity ratio to nucleus signal intensity (*n* = 5). Data are presented as protein immunofluorescent sphere area to nucleus sphere area. Values are mean ± SEM, *P* < 0.05*

Immunofluorescent pictures suggest that there is a difference in protein expression of p16 (INK4a) and p21 (Cip1) between C and HF groups (Fig. 4a). Consistent with mRNA expression, protein level of p16 (INK4a) increased significantly in livers of rats in HF group when compared to the C group, while protein level of p21 (Cip1) was significantly lower in HF group than in C group (Fig. 4b, c).

### Chromatin modifications in p16(INK4a) and p21(Cip1) genes

**Fig. 5:**
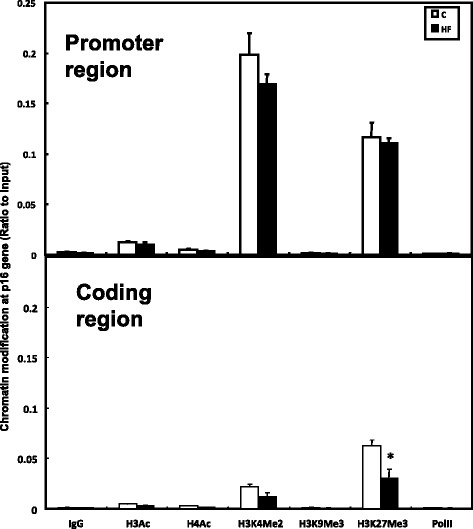
Chromatin modification at coding region and promoter region in p16(INK4a) gene in livers of rats fed C and HF diets respectively. Chromatin modification on p16(INK4a) gene at different regions in HF (*n* = 10) and C (*n* = 10) rats as determined by chromatin immunoprecipitation (ChIP) assay. Data are presented in arbitrary units as relative DNA abundance normalized to DNA of input abundance. Values are means ± SEM, *P* < 0.05*

ChIP assay was performed to investigate the possible chromatin modifications occurring in the livers of C and HF adult male rats that affected gene transcription. Results showed that methylation level of histone H3 lysine 27 (H3K27Me3) was significantly lowered in the p16(INK4a) coding region in HF rats compared with C rats. Lowered methylation level of histone H3 lysine 4 (H3K4Me2) was also observed at promoter region and in the coding region of p16(INK4a) in HF rats compared with C rats; however, no significance was detected (Fig. 5).

**Fig. 6:**
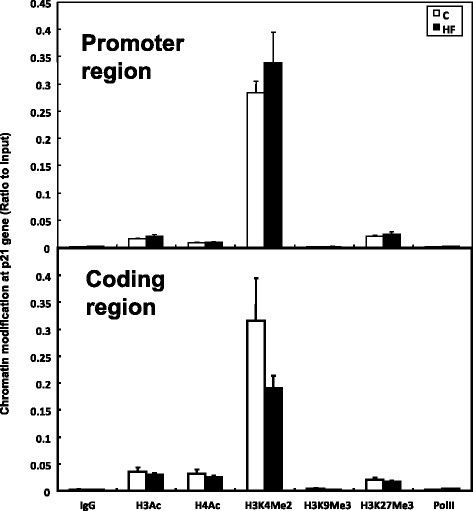
Chromatin modification at coding region and promoter region in p21(Cip1) gene in livers of rats fed C and HF diets respectively. Chromatin status in p21(Cip1) gene at different regions in HF and C rats as determined by ChIP assay. Data are presented in arbitrary units as relative DNA abundance normalized to DNA of input abundance. Values are means ± SEM

On the other hand, as for gene p21(Cip1), the methylation level of histone H3 lysine 4 (H3K4Me2) was increased at the promoter region in HF rats compared with C rats, while decreased in the coding region, with no significance detected. Meanwhile, the methylation level of histone H3 lysine 27 (H3K27Me3) was almost same at the promoter region and in the coding region of p21(Cip1) in HF rats compared with C rats (Fig. 6).

## Discussion

The purpose of the present study was to determine the mRNA expressions of CKIs p16(INK4a) and p21(Cip1) genes and to investigate the chromatin modification in these two genes induced by HF diet in livers of adult male rats and their effects on inducing hepatic physiological changes. Here, we reported that significantly increased mRNA expression of p16(INK4a) and significantly decreased p21(Cip1) mRNA expression were observed in livers of adult rats fed HF diet, comparing to rats fed C diet. The protein levels of p16 (INK4a) and p21 (Cip1) were confirmed by immunofluorescence, and the results were consistent with mRNA expression. We further investigated and discovered that expression of p16(INK4a) was regulated by chromatin modifications in the coding region. Also, we reported that HF diet induced physiological outcomes in adult rats including significantly increased energy intake and body weight, as well as significantly increased liver TAG concentration, which induced fatty liver in adult rats.

Lipid accumulation has been confirmed to be associated with fatty liver development and prevention [32]. In previous studies, an increased liver TAG concentration in animal models fed HF diet or in their offspring [33–35] was observed, as well as increased lipid accumulation (indicated by larger lipid droplets from ORO staining in animals fed HF diet) and extensive ballooning degeneration [36, 37], which is consistent with our results, indicating that HF diet induced fatty liver in rats in this current study.

We also found mRNA and protein expression of both p16(INK4a) and p21(Cip1), two well-established cell cycle inhibitors, biomarkers of aging and cellular senescence [38–40] have been altered significantly by HF diet in livers of adult rats, however, in opposite direction. The increase of p16(INK4a) mRNA can be partially explained by the decrease of tri-methylated histone H3 lysine 27 (H3K27Me3) in the coding region of p16(INK4a) that was observed in this present study, as H3K27Me3 has been considered as a suppressor of gene transcription [41–43]. On the other hand, a significantly decreased mRNA expression of p21(Cip1) was observed in HF rats; however, there was no significant difference on transcription rate of this gene between two diet groups. Further, an increased methylation level of histone H3 lysine 4 (H3K4Me2) in promoter region of p21 (Cip1) in HF rats was observed, as well as a decreased methylation level in the coding region, although no significance was detected. Consistent with our results, a declined amount of p21(Cip1)1 with an elevated expression of p16(INK4a) were observed in human diploid fibroblasts after cell achieve senescence by an early research [44]. The same research also demonstrated that p16(INK4a) and p21(Cip1) have very different age-related accumulation patterns, and the downregulation of p21(Cip1) might be a necessary part for putative differentiation program in late senescent cells. Thus, the decrease of p21(Cip1) mRNA might be explained by the differential role it plays in the mechanisms of senescence in cells. Histone H3 lysine 4 methylation (H3K4me) may also contribute to p21(Cip1) transcription [45]. Another study suggests that the absence of H3K4me2 in coding region induces increased of H3K4ac, while the degree of H3K4ac enrichment is proportional to the rate of transcription [46]. The two studies together may be used to explain the results gained from current study: in the liver of adult rats fed HF diet, a higher level of H3K4me2 in promoter region of p21(Cip1) repressed its expression; meanwhile, a lower level of H3K4me2 in coding region maintained its transcription rate, keeping it at almost the same level as in C group rats.

The regulatory mechanisms of p16(INK4a) and p21(Cip1) on cellular senescence remain unclear at this point. Previous study demonstrated that p16(INK4a) regulates cell senescence through both CDK4/6-dependent and CDK4/6-independent mechanisms. Under CDK4/6-dependent mechanism, enhanced p16(INK4a) expression disassociates and releases p21(Cip1) from cyclinD-CDK4/6 complexes, inhibiting CDK4/6 activity, and therefore promotes cell senescence and decreases regenerative capacity. However, the study also suggests that mechanism through which p16(INK4a) induces senescence varies, depending on aging condition, cell type and species [47]. Meanwhile, it is reported that lowered p21(Cip1) expression causes lower serum adiponectin, a protein that inhibits proliferation of liver cancer cells, and therefore impairs apoptosis and/or induces cell cycle progression in the liver [48, 49]. On the other hand, an enhanced p21(Cip1) expression in the liver is usually linked to accelerated hepatocyte senescence [50–52].

## Conclusion

Overall, our study demonstrates that, in adult rats fed HF diet, it activates cellular senescence through interplay between p16(INK4a) and p21(Cip1) in the liver. This is accompanied by elevated TAG accumulation. The increased expression of p16(INK4a) was associated with histone modifications, in particular, the trimethylation of histone 3 lysine 27 and the demethylation of histone 3 lysine 4, happened in promoter and coding regions respectively.

## Abbreviations

C: Control
CDK: Cyclin-dependent kinase
ChIP: Chromatin immunoprecipitation
CKI: CDK inhibitor
H&E: Hematoxylin and eosin
H3K27Me3: Trimethylation histone H3 lysine 27
H3K4: Histone H3 lysine 4
H3K4Me2: Demethylation histone H3 lysine 4
H3K9: Histone H3 lysine 9
HF: High fat
HFD: High-fat diet
NEFA: Non-esterified fatty acid
ORO: Oil red O
qPCR: Quantitative real-time polymerase chain reaction
Rb: Retinoblastoma
TAG: Triacylglycerol
TSA: Trichostatin A
